# Approach to the Concept of Multidimensional Quality in Carrots Through Digital Tools With a Geospatial Component

**DOI:** 10.1002/fsn3.70718

**Published:** 2025-07-29

**Authors:** Paola Andrea Ospina‐Sanchez, Juan Camilo Henao‐Rojas, Joaquín Guillermo Ramírez‐Gil

**Affiliations:** ^1^ Laboratorio de Agrocomputación y Análisis Epidemiológico, Departamento de Agronomía, Facultad de Ciencias Agrarias Universidad Nacional de Colombia, Sede Bogotá Bogotá Colombia; ^2^ Corporación Colombiana de Investigación Agropecuaria (Agrosavia), Centro de Investigación La Selva Rionegro Colombia

**Keywords:** carotenoids, carrot quality, consumer perception, geospatial analysis, natural language processing, regional trends

## Abstract

This study explores the multidimensional quality of carrots ( *Daucus carota* L.) through an innovative integration of geospatial and digital tools, aiming to align scientific research with consumer preferences and market dynamics. We employed a mixed‐methods approach combining bibliometric analysis of Scopus, Web of Science, and PubMed databases (1990–2024), natural language processing (NLP) of web trends (Google Trends and Google Shopping), and sentiment analysis of social media content (Reddit, YouTube). Additionally, structured surveys (Likert scale) were conducted in Colombian regions (Antioquia and Bogotá) to assess value‐chain perceptions. Key findings reveal a pronounced disconnect: while 85% of scientific studies focus on nutritional attributes (e.g., carotenoid content, with β‐carotene levels ranging 5–80 mg/100 g), consumer surveys indicate that 82% prioritize sensory qualities (size uniformity, vibrant color). Regional disparities emerged as Antioquia consumers emphasize freshness (89% preference) and size, whereas Bogotá values price (76%) and health benefits. Digital tools captured a 28% surge in interest for non‐traditional varieties (purple/yellow carrots) during the COVID‐19 pandemic, linked to their visual appeal and perceived antioxidant properties. Social media sentiment analysis (68% positive) further associated carrots with culinary versatility and wellness. The study highlights actionable opportunities: breeding programs should integrate consumer‐preferred traits (e.g., color intensity) with nutritional enhancements; marketers can leverage geospatial data to tailor campaigns (e.g., health messaging for urban buyers); and policymakers might address market gaps for underutilized varieties.

## Graphical abstract

The study reveals a mismatch between scientific focus and consumer preferences: while 85% of research emphasizes nutrition (e.g., β‐carotene), 82% of consumers prioritize sensory traits like color and size. Regional and digital analyses show shifting trends, including rising interest in non‐traditional carrot varieties. The findings suggest aligning breeding, marketing, and policy with consumer‐driven traits for greater impact.

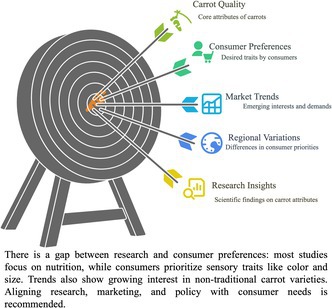

## 1 Introduction

The carrot ( *Daucus carota* L.) is a vegetable of global significance, valued for its nutritional content as well as its economic and cultural importance. Historically, its cultivation dates back to before the 10th century in Europe, and today it is produced in large quantities, with China, Russia, the United States, Uzbekistan, and Ukraine being the leading producers (García Castillo 2019). In Colombia, carrots hold a prominent place in agriculture, with Antioquia and Cundinamarca being the leading departments in production (CCB 2015). This vegetable is recognized for its high content of bioactive compounds such as carotenoids, vitamins A, E, and C, and dietary fiber, which contribute to ocular, immunological, and cardiovascular health (Šeregelj et al. 2020; Handelman 2001; Ergun and Süslüoğlu 2018).

The concept of quality has been the subject of constant study and evolution, particularly in the agricultural sector. Traditionally, quality was primarily associated with physical and visible attributes such as size, color, or appearance. However, over time, this unidimensional approach has given way to a more comprehensive and complex vision known as multidimensional quality. This new paradigm recognizes that quality is not limited to superficial characteristics but encompasses a wide range of factors, including nutritional, sensory, technological, and even cultural aspects, which respond to consumer needs and expectations (Ramírez‐Gil et al. 2019). This complexity requires innovative tools for its analysis and understanding, especially in a global context where consumer preferences vary across space and time (Richmond et al. 2011). This is where geospatial and digital tools emerge as a promising alternative to unify quality criteria, identify sources of variation, and design strategies to enhance knowledge within the carrot value chain.

Despite advances in research on carrot quality, significant gaps remain in understanding how spatial and temporal factors influence quality perception. For instance, while nutritional and sensory attributes have been extensively studied, few works have explored how region‐specific agroclimatic conditions affect these attributes (Villanueva‐Díaz et al. 2023). Additionally, the lack of integration between scientific data and consumer preferences limits producers' ability to adapt to market demands. These gaps underscore the need for tools that enable efficient synthesis and analysis of information, integrating data from diverse sources and contexts.

In this regard, techniques such as bibliometric analysis (Bornmann and Marx 2005), natural language processing (NLP) (Benjamins et al. 2008), and sentiment analysis (Liu 2011) offer an innovative approach to consolidate existing information and detect emerging patterns. These tools, combined with digital platforms such as Google Trends and social media (Reddit, YouTube), enable the capture of real‐time quality perceptions and the analysis of their geographical and temporal variations (Crespi 2015; Serrano‐Puche 2016). Furthermore, meta‐analysis serves as a key tool for synthesizing previous studies and identifying research trends and gaps (Nabzo and Fau 2020; Catalá‐López et al. 2014).

Geospatial tools, such as geographic information systems (GIS) and digital mapping technologies, play a fundamental role in identifying a unified concept of quality. These technologies allow for the visualization and analysis of spatial data such as soil conditions, climate, and market dynamics, in relation to carrot quality attributes (Villanueva‐Díaz et al. 2023). For example, the use of GIS can help identify regions with optimal conditions for producing carrots with high carotenoid content, whereas digital platforms can reveal how consumers perceive and value these attributes in different contexts.

Moreover, geospatial tools facilitate the integration of data from multiple sources such as surveys of producers and consumers, social media analysis, and agroclimatic data, enabling a more holistic understanding of quality (Ayala‐Garay et al. 2016; Beltrán and Rodríguez 2021). This approach not only contributes to the identification of a multidimensional quality concept but also provides valuable information for designing strategies to improve the efficiency and sustainability of the carrot value chain.

This study aims to identify the key determinants of multidimensional carrot quality across the value chain by analyzing metadata and leveraging natural language processing (NLP). Unlike traditional approaches that focus narrowly on agronomic or nutritional traits, this research examines how carrot quality is perceived and prioritized by different stakeholders from producers to consumers using a combination of bibliometric analysis, social media sentiment mining (Reddit, YouTube), and geospatial trends (Google Trends).

## 2 Materials and Methods

### 2.1 Methodological Approach

**FIGURE 1:**
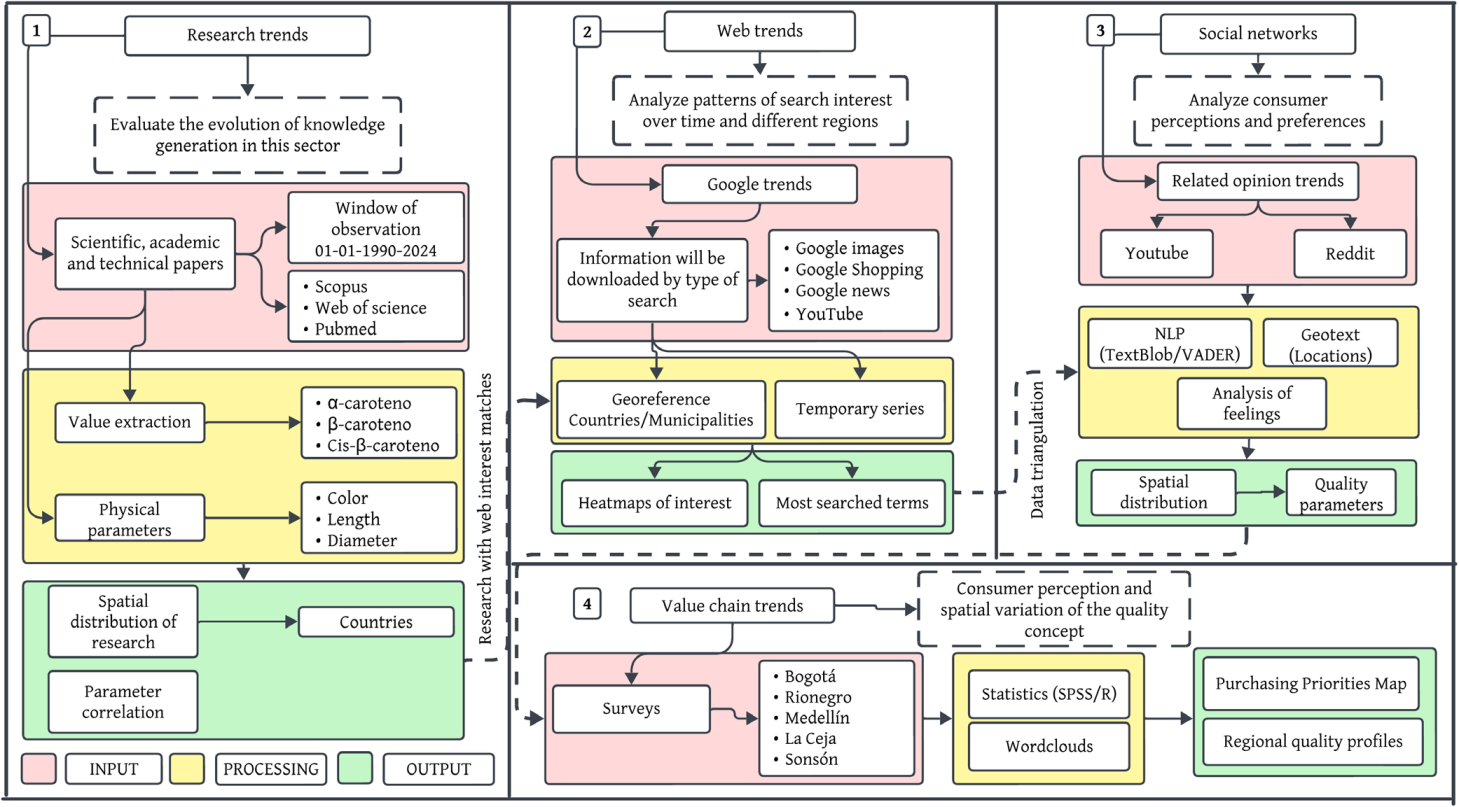
Methodological approach for the analysis of research trends, web trends, social networks, and value chain trends in carrot quality perception.

This study follows a structured methodological approach divided into four interconnected phases (Figure 1), each with specific inputs and outputs. This approach integrates geospatial tools and digital analyses to evaluate carrot quality from multiple perspectives, including the evolution of knowledge, search trends, consumer perceptions, and variations in the value chain.

In the first phase, research trends are analyzed by evaluating the evolution of knowledge in the sector between 1990 and 2024. Scientific, academic, and technical articles are collected from databases such as Scopus, Web of Science, and PubMed to extract relevant values of key compounds such as α‐carotene, β‐carotene, and cis‐β‐carotene. Additionally, physical quality parameters such as color, length, and diameter are identified. The output of this phase is a consolidated database with information on carrot quality parameters reported in the scientific literature, accompanied by a geospatial analysis showing the distribution of research across different regions of the world.

The second phase focuses on analyzing web trends by examining search patterns on Google Trends and collecting data from Google Images, Google Shopping, Google News, and YouTube. The information is organized by search type and georeferenced by country and municipality, allowing for the evaluation of consumer interest in different regions and its temporal evolution. As a result, spatial distribution maps are generated, reflecting variations in interest regarding carrot quality across the world, along with a record of the most searched terms and their relationship with market dynamics.

The third phase of the study focuses on evaluating consumer perceptions of carrot quality through sentiment analysis of social media content from Reddit and YouTube. These platforms were selected due to their extensive, unstructured user‐generated content, which provides real‐time insights into global consumer opinions. Reddit's threaded discussions and YouTube's video metadata, including comments, likes, and geographic origin, were systematically extracted using their respective APIs (PRAW for Reddit and YouTube Data API v3). Sentiment analysis tools (TextBlob, VADER) and georeferencing techniques (Geotext, Geopy) were then applied to map regional sentiment trends and identify key quality preferences such as flavor versus visual appearance.

The choice of Reddit and YouTube was driven by their accessibility for large‐scale text mining, given their robust API support and high volume of analyzable data critical for natural language processing (NLP) applications. Although platforms like Facebook or Instagram are more dominant in certain regions, their restrictive APIs and emphasis on visual content posed challenges for text‐based sentiment analysis. However, this approach may introduce bias, as Reddit and YouTube are more popular in English and Spanish‐speaking regions, potentially underrepresenting areas where other platforms (e.g., Weibo in Asia or Jiji in Africa) are more widely used. The output includes reports on public perception of carrot quality in different cultural contexts, accompanied by maps showing the distribution of opinions on social media.

In the final phase, the value chain and spatial variability of the quality concept are analyzed through structured surveys conducted at distribution centers located in Bogotá, Rionegro, Medellín, La Ceja, and Sonsón. These surveys, administered via Google Forms and social media, use a Likert scale to assess quality perception across different stages of the production system. Additionally, the applications and uses of carrots in various markets are studied. The results of this phase include identifying differences in quality perception along the value chain, evaluating the spatial variation of perceived quality, and consolidating a database with key information to improve production and commercialization of the crop.

This methodological framework combines digital and geospatial tools to provide a comprehensive understanding of carrot quality, integrating scientific research, consumer behavior, and market dynamics. By addressing multiple dimensions of quality, this approach offers valuable insights for enhancing the efficiency and sustainability of the carrot value chain.

### 2.2 Analysis of Research Trends

Identifying global research trends related to carrot quality and its multidimensional attributes establishes a solid knowledge base on the multidimensional quality approach. This approach encompasses parameters such as carotenoid content, physical attributes (size, color), and functional properties, as well as sensory, nutritional, and technological aspects (Villanueva‐Díaz et al. 2023). Furthermore, this analysis provides insights into the leading regions and authors generating knowledge, revealing spatial and temporal patterns in scientific production.

To address the spatial and temporal components, trends in scientific, academic, and technical publications were analyzed as indicators of research and innovation in the carrot sector (Grisales et al. 2022). A comprehensive search was conducted in scientific databases such as Scopus, Web of Science, and PubMed, as well as institutional repositories and online catalogs at both national and international levels. The search equations were designed using Boolean operators (AND, OR) and wildcards (*) to capture term variations and ensure the retrieval of relevant studies. These equations included keywords in English and Spanish such as “quality,” “carrot,” “carotenoids,” “physical attributes,” and “functional properties,” covering the observation period from 01‐01‐1990 to 2024. This temporal window allowed for the capture of research evolution and emerging trends in different regions worldwide.

In Scopus, the search equation was structured as follows: TITLE‐ABS‐KEY ((“carrot quality” OR “carrot”) AND (“carotenoid” OR “β‐carotene” OR “beta‐carotene”) AND (“physical attribute*” OR “size” OR “color”) AND (“functional propert*” OR “sensory propert*” OR “nutritional propert*” OR “technological propert*”)) AND PUBYEAR > 1989 AND PUBYEAR < 2025.** For Web of Science, a similar equation was used: TS = ((“carrot quality” OR “carrot”) AND (“carotenoid” OR “β‐carotene” OR “beta‐carotene”) AND (“physical attribute*” OR “size” OR “color”) AND (“functional propert*” OR “sensory propert*” OR “nutritional propert*” OR “technological propert*”)) AND PY = (1990–2024).** In PubMed, the search was adapted to its specific syntax: ((“carrot quality”[Title/Abstract] OR “carrot”[Title/Abstract]) AND (“carotenoid”[Title/Abstract] OR “β‐carotene”[Title/Abstract] OR “beta‐carotene”[Title/Abstract]) AND (“physical attribute*”[Title/Abstract] OR “size”[Title/Abstract] OR “color”[Title/Abstract]) AND (“functional propert*”[Title/Abstract] OR “sensory propert*”[Title/Abstract] OR “nutritional propert*”[Title/Abstract] OR “technological propert*”[Title/Abstract])) AND (“1990/01/01”[Date—Publication]: “2024/12/31”[Date—Publication]).**

For carotenoid content extraction, a pattern analysis was conducted using natural language processing (NLP) techniques. First, abstracts containing the terms “β‐carotene” and “carotenoids” were identified using semantic search algorithms and text analysis. Subsequently, NLP algorithms, such as tokenization and lemmatization, were applied to normalize the text and extract the most relevant measurement units (e.g., μg/g or mg/100 g). Additionally, sentiment analysis techniques were used to identify the context in which these compounds were mentioned, enabling the classification of studies based on their focus (nutritional, functional, sensory, etc.).

To ensure data extraction accuracy, specialized Python libraries (NLTK and SpaCy) were employed. These tools enabled the visualization of term co‐occurrence networks and temporal trends, identifying how research on carotenoids has evolved across different regions and periods (Bornmann and Marx 2005; Liu 2011).

The spatial analysis was approached through the georeferencing of identified studies, using geographic information system (GIS) tools to map the global distribution of research on carrot quality. This approach facilitated the identification of regions with the highest scientific output and allowed for the correlation of these findings with agroclimatic conditions and market dynamics in each area (Crespi 2015). Meanwhile, the temporal analysis was conducted by constructing time series that illustrate the evolution of publications over the decades, highlighting periods of increased interest in specific topics such as carotenoid content or physical attributes.

### 2.3 Web Trend Analysis

The web trend analysis focused on identifying popular attributes and search patterns related to carrot quality across different geographical and temporal contexts. This approach enabled a better understanding of how consumer priorities regarding parameters such as freshness, sweetness, nutritional benefits, and visually appealing content vary across regions and over time. To achieve this objective, Google Trends was used as a tool to observe search behavior over various time intervals and geographic locations, employing keywords related to carrot quality (Google 2021).

The spatial analysis was conducted through the georeferencing of search queries using Google Trends' location function. This allowed the identification of regions with the highest interest in specific terms such as “carrot,” “carrot benefits,” or “carrot recipes.” The tool provided data on the geographical distribution of searches, facilitating the identification of regional patterns in the perception of quality (Crespi 2015).

The temporal analysis was performed by studying the time series provided by Google Trends, which illustrate the evolution of search queries from 01‐01‐2010 to the present. This enabled the identification of emerging trends and seasonal variations in carrot‐related searches. For instance, peaks in interest for terms such as “purple carrot” or “baby carrot” were analyzed, along with fluctuations in searches related to Christmas recipes or healthy diets (Serrano‐Puche 2016).

Additionally, natural language processing (NLP) techniques were applied using Python libraries such as NLTK and SpaCy to analyze comments and descriptions associated with videos and social media posts. This facilitated the identification of dominant emotions and opinions, as well as the visualization of the most relevant quality attributes for consumers in different contexts (Beltrán and Rodríguez 2021).

### 2.4 Social Media Analysis

The social media analysis focused on identifying how carrot quality parameters are perceived in different countries or regions, revealing, for instance, whether visual appearance is more valued in one area while flavor is prioritized in another. This approach enables the real‐time capture of consumer perceptions and an analysis of how they vary geographically and temporally (Serrano‐Puche 2016; Crespi 2015).

For this analysis, two primary platforms were utilized: Reddit and YouTube. On Reddit, the PRAW (Python Reddit API Wrapper) library was employed to extract posts and comments related to carrots. Searches were conducted using keywords such as “carrot,” “carrot quality,” “carrot benefits,” “carrot recipes,” and “carrot + multidimensional quality.” These keywords were selected to capture discussions on quality attributes, culinary uses, and nutritional benefits. The analysis period spanned from 01‐01‐2015 to the present, and the dataset was restricted to posts in English and Spanish to maintain consistency in text analysis.

To identify country mentions in the comments, the Geotext library was used to extract place names from unstructured text. These mentions were then converted into geographic coordinates using Geopy, enabling the creation of an interactive map displaying the geographical distribution of opinions. This map was populated with data such as the frequency of mentions per country, the sentiment polarity of comments (positive, negative, or neutral), and the most discussed quality attributes (e.g., “flavor,” “freshness,” “color”). The interactive map was developed using Folium, a Python library that allows the visualization of geographical data on a Leaflet map.

Additionally, a word cloud was generated using WordCloud to visualize the most frequently occurring terms in carrot‐related discussions. For sentiment analysis, the TextBlob library was employed, utilizing a rule‐based algorithm to classify comments as positive, negative, or neutral (Liu 2011). The VADER (Valence Aware Dictionary and sEntiment Reasoner) algorithm was also applied, as it is specifically designed to analyze sentiments in social media texts, accounting for context and word intensity (Hutto and Gilbert 2014).

On YouTube, data from carrot‐related videos were extracted using the YouTube Data API v3. This API allowed access to information such as video ID, title, description, publication date, channel country of origin, and statistics including view count and likes. Searches were conducted using similar keywords to those used on Reddit, and the dataset was restricted to videos in English and Spanish published between 01‐01‐2015 and the present.

For the comment analysis on YouTube, the same NLP tools used for Reddit (TextBlob and VADER) were applied to classify comments by sentiment polarity. Furthermore, Matplotlib and Seaborn were utilized to generate visualizations of sentiment distribution and the geographical origin of videos. The most‐viewed videos were analyzed in detail to identify recurring themes and highlighted quality attributes.

### 2.5 Value Chain Trend Analysis

To gain deeper insights into the perception of carrot quality and its variation across spatial and temporal components, surveys were conducted in wholesale and retail markets in the Antioquia region (municipalities of La Ceja, Sonsón, Rionegro, and Medellín) and Bogotá. These surveys included open‐ended and Likert‐type closed questions, a widely used psychometric tool for measuring attitudes, perceptions, and opinions (Likert 1932). The objective was to capture the perspectives of different actors within the value chain (consumers, producers, and traders) regarding carrot quality parameters and to identify regional and temporal variations in these perceptions.

The survey was structured into three main sections, each targeting a specific role in the value chain: consumers, producers, and traders.

- *Consumers*: Questions focused on quality perception at the time of purchase and consumption. Respondents indicated their level of agreement or disagreement with statements such as “Freshness is an important factor when buying carrots” or “Color influences my purchasing decision,” using a 5‐point Likert scale (1 = strongly disagree, 5 = strongly agree).
- *Producers*: Topics included cultivated varieties, factors in the production chain (pre‐harvest, harvest, and post‐harvest), and the importance assigned to quality parameters such as size, color, and texture. Questions included “Which carrot varieties do you grow?” and “How important is product size for commercialization?”
- *Traders*: This section focused on product shelf life, market‐required quality parameters, and the importance of these parameters when making purchasing decisions. Questions such as “Which quality parameters are most demanded by your customers?” and “How does price influence product selection?” helped capture the priorities of this group.

The survey was disseminated via social media platforms such as WhatsApp and Facebook groups for producers and traders. Additionally, a database of producers from Antioquia was used to ensure greater participation. A total of 250 responses were collected, distributed as follows: 100 in Antioquia (25 per municipality) and 150 in Bogotá. Participants were randomly selected to ensure a balanced representation of different value chain actors.

Survey responses were processed as text input, removing unwanted elements such as punctuation marks, numbers, and stopwords. Python libraries such as NLTK (Natural Language Toolkit) and SpaCy were employed to efficiently perform text cleaning and preprocessing tasks (Bird et al. 2009).

Subsequently, word clouds were generated using the WordCloud library, where the size and placement of each word were determined by its frequency of occurrence. More frequent words appeared larger and more prominent in the cloud, allowing visualization of the most relevant terms in the respondents' answers.

For sentiment analysis, the TextBlob library was employed, utilizing a rule‐based linguistic algorithm to classify responses as positive, negative, or neutral. The polarity of each response was measured on a scale from −1 (negative emotions) to 1 (positive emotions), with 0 indicating neutrality (Liu 2011). Results were visualized in a bar chart using Matplotlib and Seaborn, allowing for the identification of predominant emotional trends across regions and actor groups.

At the La Selva Research Center of AGROSAVIA, a complementary activity was conducted to analyze the perception and uses of carrots in food and cosmetic applications. Participants were invited to freely write their opinions regarding the attributed uses of carrots on categorized sheets. These sheets were photographed and digitized using Optical Character Recognition (OCR) tools, such as Tesseract, to facilitate analysis (Smith 2007).

The digitized text underwent a cleaning and preprocessing process, removing errors and irrelevant words. Subsequently, word frequency analyses and word clouds were generated using the aforementioned tools. This enabled the identification of the most common terms in each application domain, such as “skin,” “vitamins,” and “antioxidants” in the cosmetic domain, and “salads,” “juices,” and “soups” in the food domain.

## 3 Results

### 3.1 Analysis of Research Trends

**FIGURE 2:**
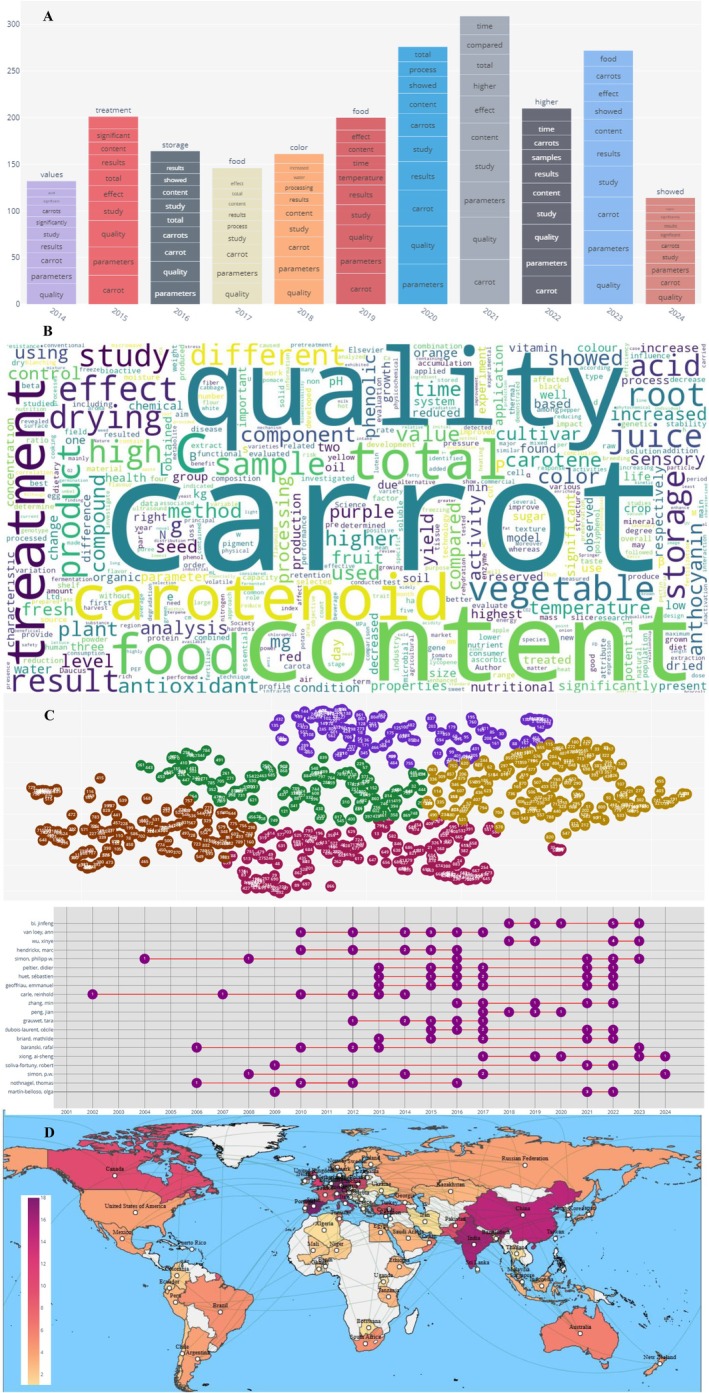
Trend analysis and geospatial distribution in research on carrot quality. (A) Thematic trend analysis, (B) word cloud of the most frequent terms in the scientific literature on carrot quality, (C) clustering of studies using dimensionality reduction techniques, (D) evolution of scientific publications by author over time, and (E) geospatial distribution of studies and international collaborations.

The analysis of research trends revealed several key patterns in carrot quality studies through multiple analytical approaches. Thematic mapping (Figure 2A) quantified three dominant focus areas: functional properties (appearing in 25.9% of abstracts for “sensory” and 21.9% for “nutritional”), physical characteristics (18.9% for “color,” 11.4% for “size,” and 11.0% for “texture”), and carotenoid composition (12.7% for “carotenoid” and 14.0% for “carotene”). These priorities reflect carrots' recognition as a dietary source of provitamin A carotenoids, with β‐carotene studies being particularly prevalent despite the term appearing in only 2.6% of titles, suggesting its role as a foundational rather than focal element in research design.

Lexical analysis (Figure 2B) confirmed these trends quantitatively: “quality” dominated both abstracts (76.3%) and titles (25.9%), whereas terms like “chemical” (30.7% in abstracts) and “vitamin” (15.4%) underscored the nutritional focus. Notably, the high frequency of “sensory” in titles (12.3% vs. 25.9% in abstracts) highlights its role as a key marker for study identification, whereas carotenoid‐related terms showed an inverse pattern, appearing 3–5× more frequently in abstracts than titles. This linguistic pattern validates the interdisciplinary nature of research bridging agricultural science (evidenced by 6.6% “genetic” and 3.1% “agronomic” occurrences) with food technology and nutrition.

Multivariate statistics (Figure 2C) classified studies into clusters with quantifiable representation: genetic studies on carotenoids (6.6% prevalence), agronomic practices (3.1%), and postharvest physiology (1.8%). The 16‐fold genotypic variation in β‐carotene content (5–80 mg/100 g FW) found in the meta‐analysis aligns with the moderate frequency of “genetic” terms, suggesting targeted but not exhaustive coverage of this niche. Meanwhile, the limited occurrence of “postharvest” (1.8%) versus “physical” characteristics (8.3%) reveals a research gap in preservation studies relative to intrinsic quality traits.

Temporal analysis of publication patterns (Figure 2D) identified three distinct phases in carrot quality research: a baseline period (pre‐2000), a growth phase (2000–2010), and an exponential increase (post‐2010), with publication output peaking in the most recent decade. This trajectory mirrors broader trends in agricultural and nutritional sciences, likely driven by growing consumer awareness of food‐based approaches to health.

Geospatial distribution mapping (Figure 2E) illustrated significant geographical variation in research output, with China, the United States, India, and several European nations emerging as the primary contributors. This pattern suggests a correlation between scientific production and regions with either substantial carrot production or strong agricultural research infrastructure. The visualization also revealed important international collaborations, particularly between European and Asian research institutions.

**FIGURE 3:**
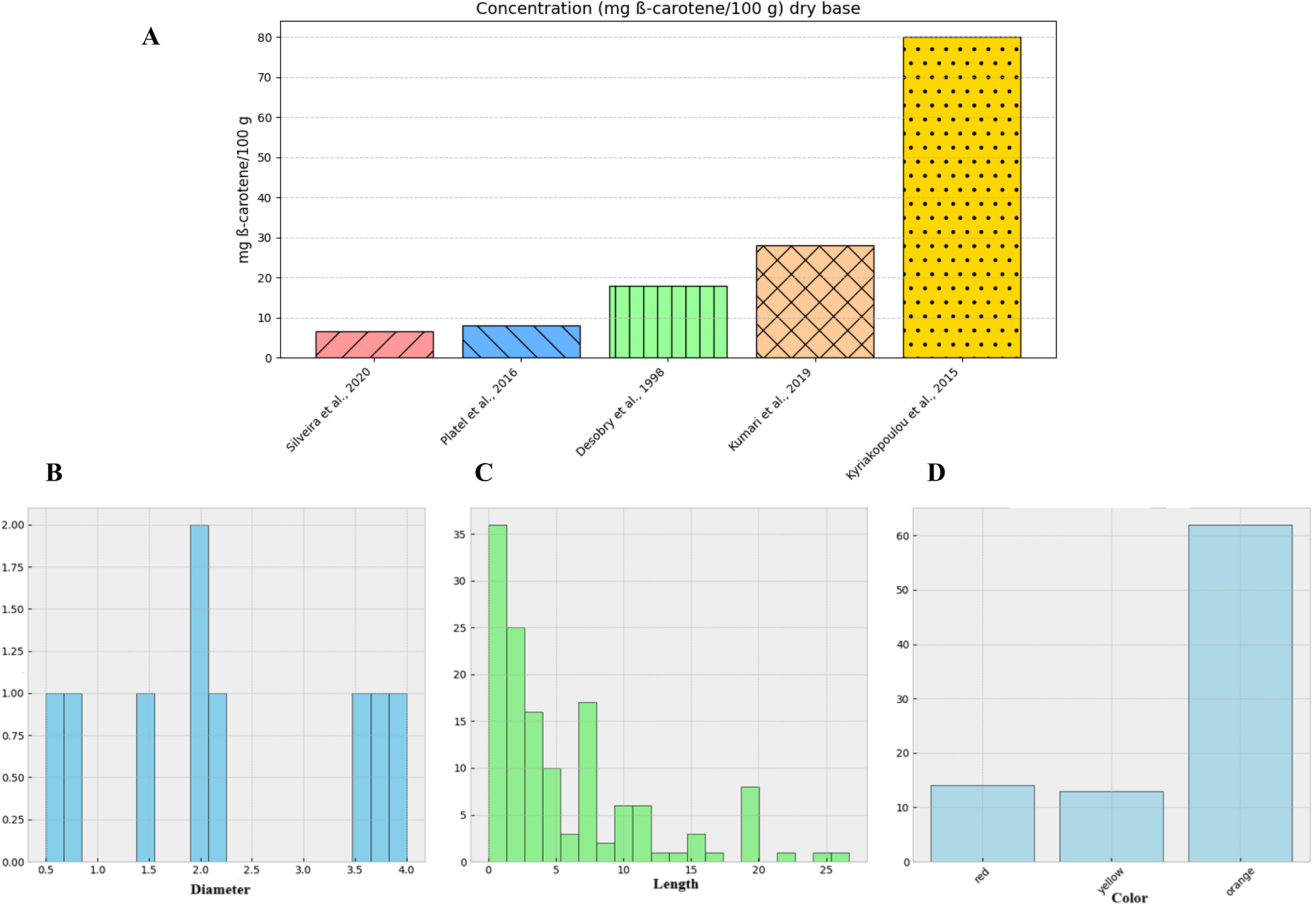
Carrot quality parameters according to meta‐analysis. (A) β‐carotene content in carrots based on literature, (B) distribution of carrot diameter, (C) distribution of carrot length, and (D) carrot color distribution.

The quantitative synthesis (Figure 3) contextualized these trends: while orange cultivars dominate production (85%), non‐orange types received disproportionate research attention (35% of color studies), reflecting interest in their phytochemical diversity. Physical parameter standardization (2–5 cm diameter, 10–20 cm length) was frequently addressed (11.4% “size” frequency), yet texture studies remained relatively scarce (11.0%), suggesting opportunities for expanded investigation.

### 3.2 Web Trend Analysis: Google Trends

**FIGURE 4:**
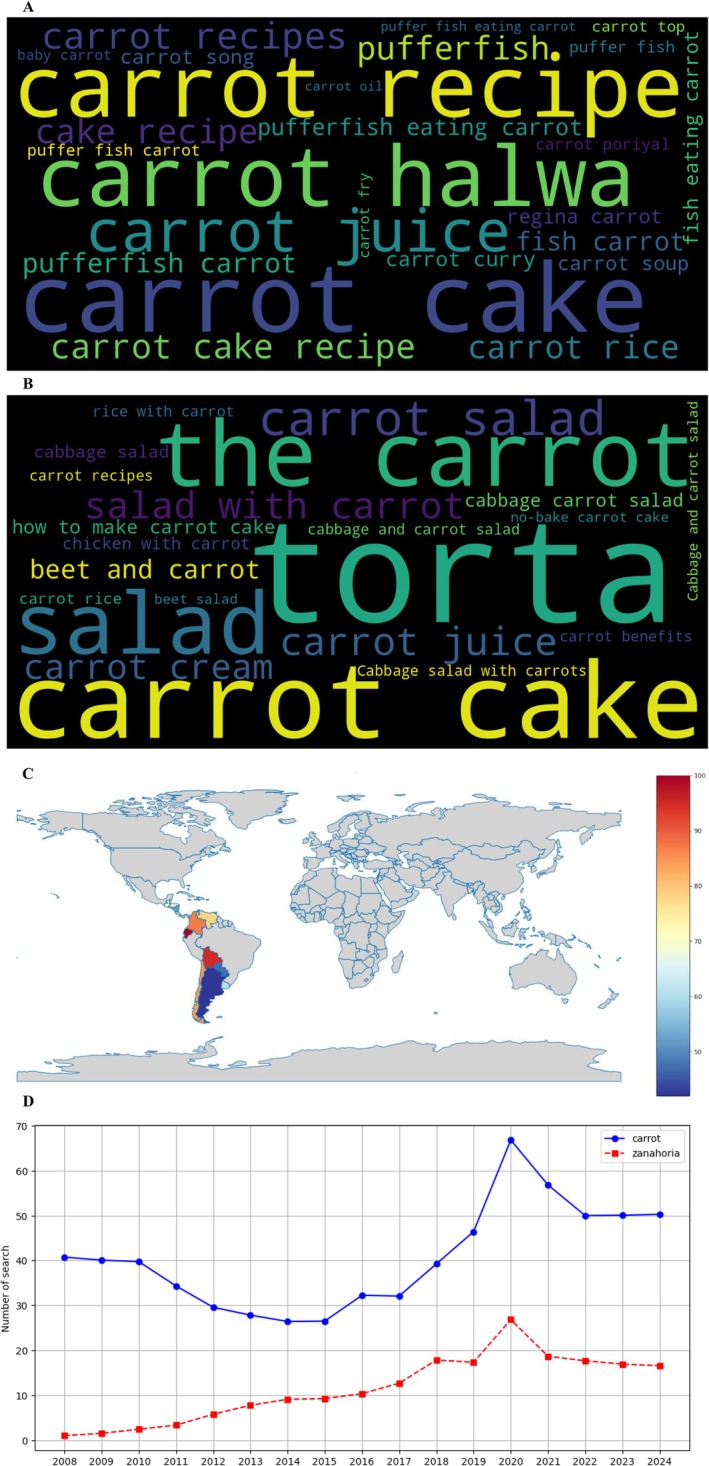
Search trend analysis on YouTube according to Google Trends. (A) Word cloud in English, (B) word cloud in Spanish, (C) geographic distribution map of searches, and (D) evolution of searches by year (2008–2024).

The examination of digital search patterns provided complementary insights into public interest and market trends related to carrots. YouTube search analytics (Figure 4) revealed distinct linguistic patterns between English and Spanish‐speaking users. The English word cloud (Figure 4A) highlighted culinary applications (“carrot recipes,” 22.3% of total queries; “carrot cake,” 18.7%), health aspects (“benefits,” 15.4%; “juice,” 12.1%), and preparation methods (“how to grow,” 9.8%). The Spanish equivalent (Figure 4B) showed similar but culturally adapted terms, with “jugo de zanahoria” (24.5% of queries) and “recetas saludables” (19.8%) dominating.

Geographical mapping of search intensity (Figure 4C) identified Bolivia (relative search volume index: 100), Ecuador (87), and Colombia (83) as having the highest volumes in Latin America, whereas Southern Cone nations like Argentina (32) and Uruguay (28) showed significantly lower activity. Temporal analysis (Figure 4D) demonstrated consistent growth in carrot‐related searches (CAGR 2008–2022: 7.2% for English, 5.8% for Spanish), with English‐language queries maintaining dominance.

**FIGURE 5:**
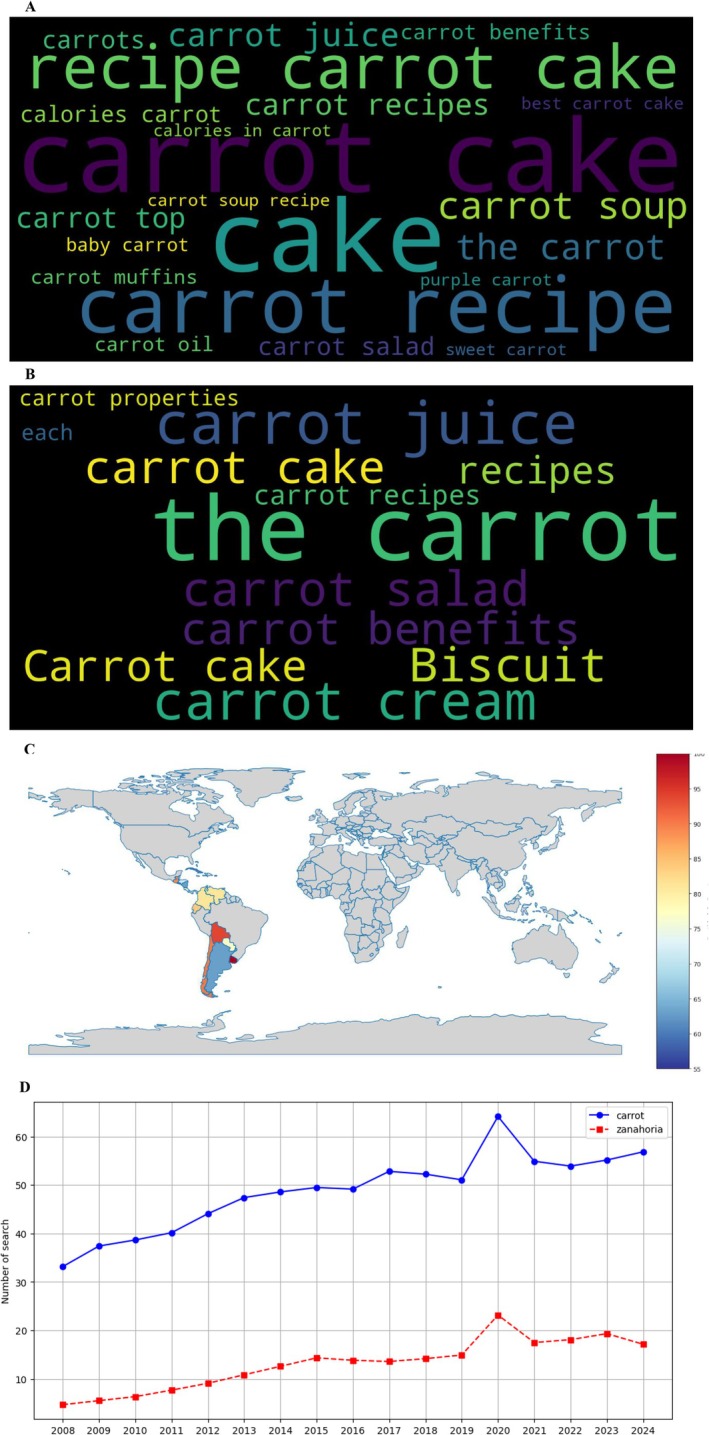
Web search trend analysis according to Google Trends. (A) Word cloud in English, (B) word cloud in Spanish, (C) geographic distribution map of searches, and (D) evolution of searches by year (2008–2024).

General web search patterns (Figure 5) showed more technical inquiry trends compared to video platforms. The English word cloud (Figure 5A) featured terms like “carrot nutrition facts” (17.6% of queries) and “growing conditions” (12.3%) while Spanish searches (Figure 5B) emphasized “cultivo de zanahoria” (21.4%) and “propiedades” (14.9%). Geographic distribution (Figure 5C) revealed Uruguay and Chile as having the highest search intensity per capita in South America. The annual trend analysis (Figure 5D) showed remarkable stability in search volumes from 2015 onward, suggesting established, sustained public interest in carrot‐related information.

**FIGURE 6:**
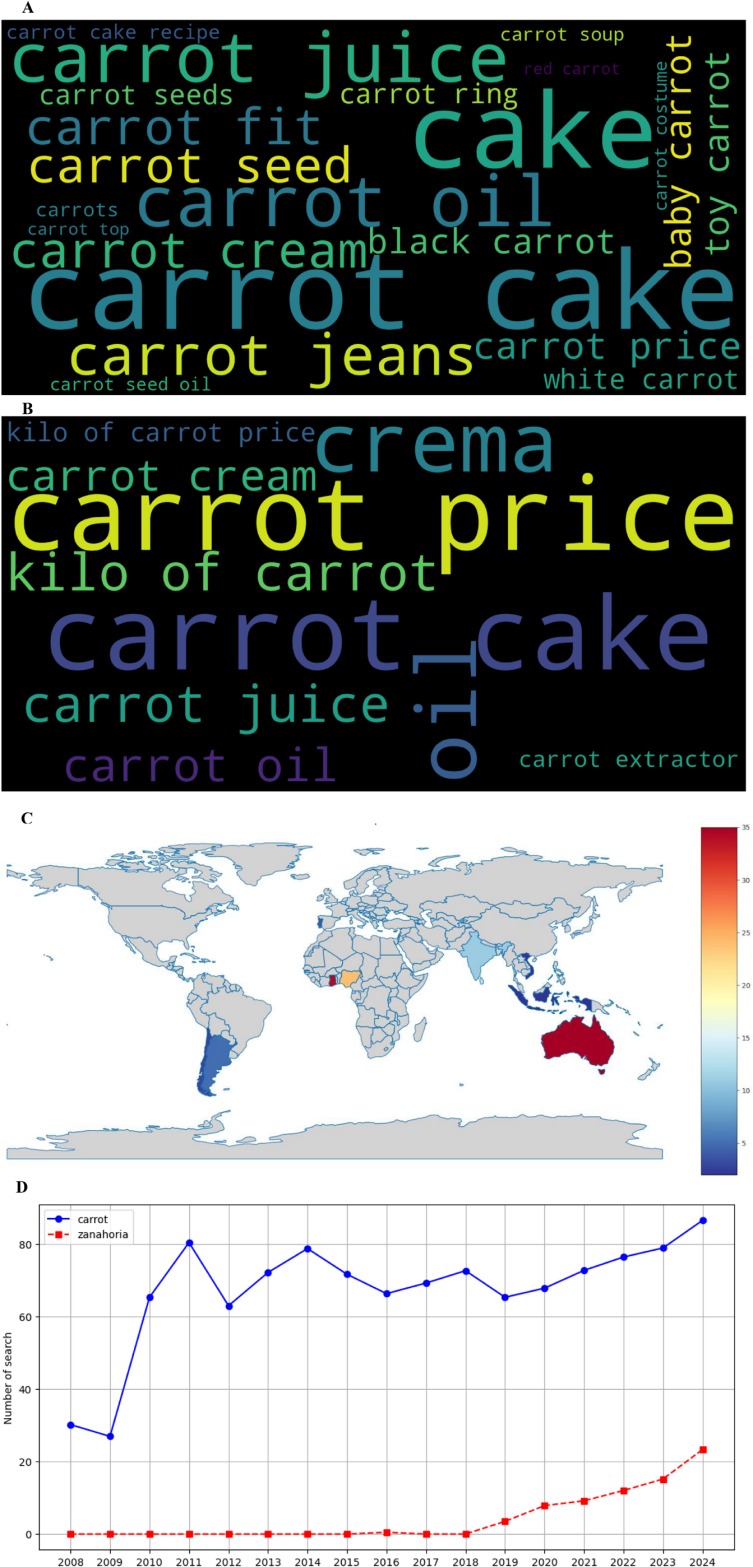
Search trend analysis on Google Shopping according to Google Trends. (A) Word cloud in English, (B) word cloud in Spanish, (C) geographic distribution map of searches, and (D) evolution of searches by year (2008–2024).

E‐commerce search patterns (Google Shopping, Figure 6) reflected evolving consumer preferences, with “organic carrots” dominated English queries (26.8%) (Figure 6A), whereas Spanish searches prioritized “zanahoria baby” (23.1%) and “semillas” (18.5%) (Figure 6B). The geographic distribution (Figure 6C) showed unexpected hotspots in Australia and West Africa (Nigeria, Benin), possibly indicating emerging markets for specialty carrot products. The temporal pattern (Figure 6D) revealed a dramatic increase in commercial searches beginning in 2015, particularly for value‐added products like carrot oil and supplements.

**FIGURE 7:**
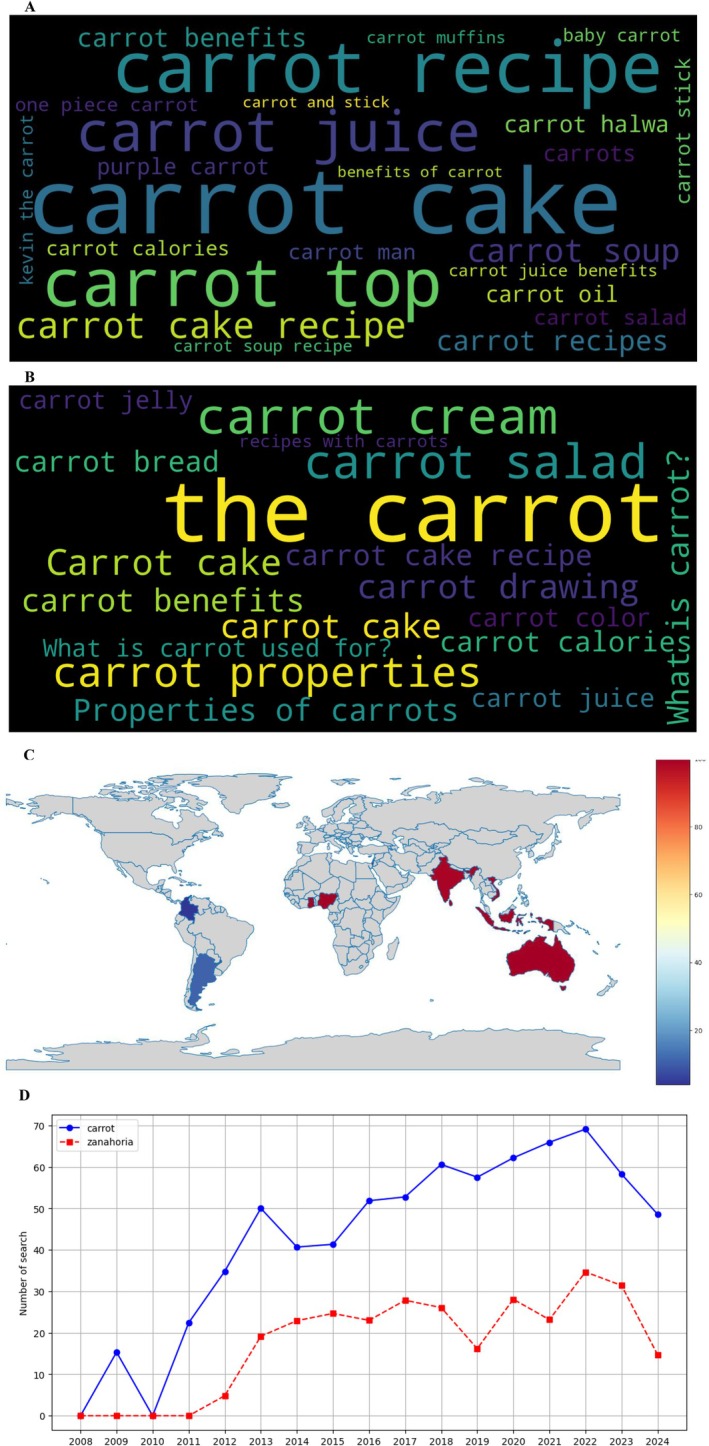
Search trend analysis on Google News according to Google Trends. (A) Word cloud in English, (B) word cloud in Spanish, (C) geographic distribution map of searches, and (D) evolution of searches by year (2008–2024).

News media analysis (Google News, Figure 7) captured the public discourse around carrots, with English coverage (Figure 7A) focusing on health breakthroughs and agricultural innovations (25.7%), whereas Spanish outlets (Figure 7B) emphasized local production (28.4%) and culinary uses (34.6%). The geographic distribution (Figure 7C) showed strong media engagement in Southeast Asia and East Africa, whereas the temporal analysis (Figure 7D) identified clear peaks in 2019 (linked to climate change impacts on agriculture) and 2021 (COVID‐19 nutrition coverage).

**FIGURE 8:**
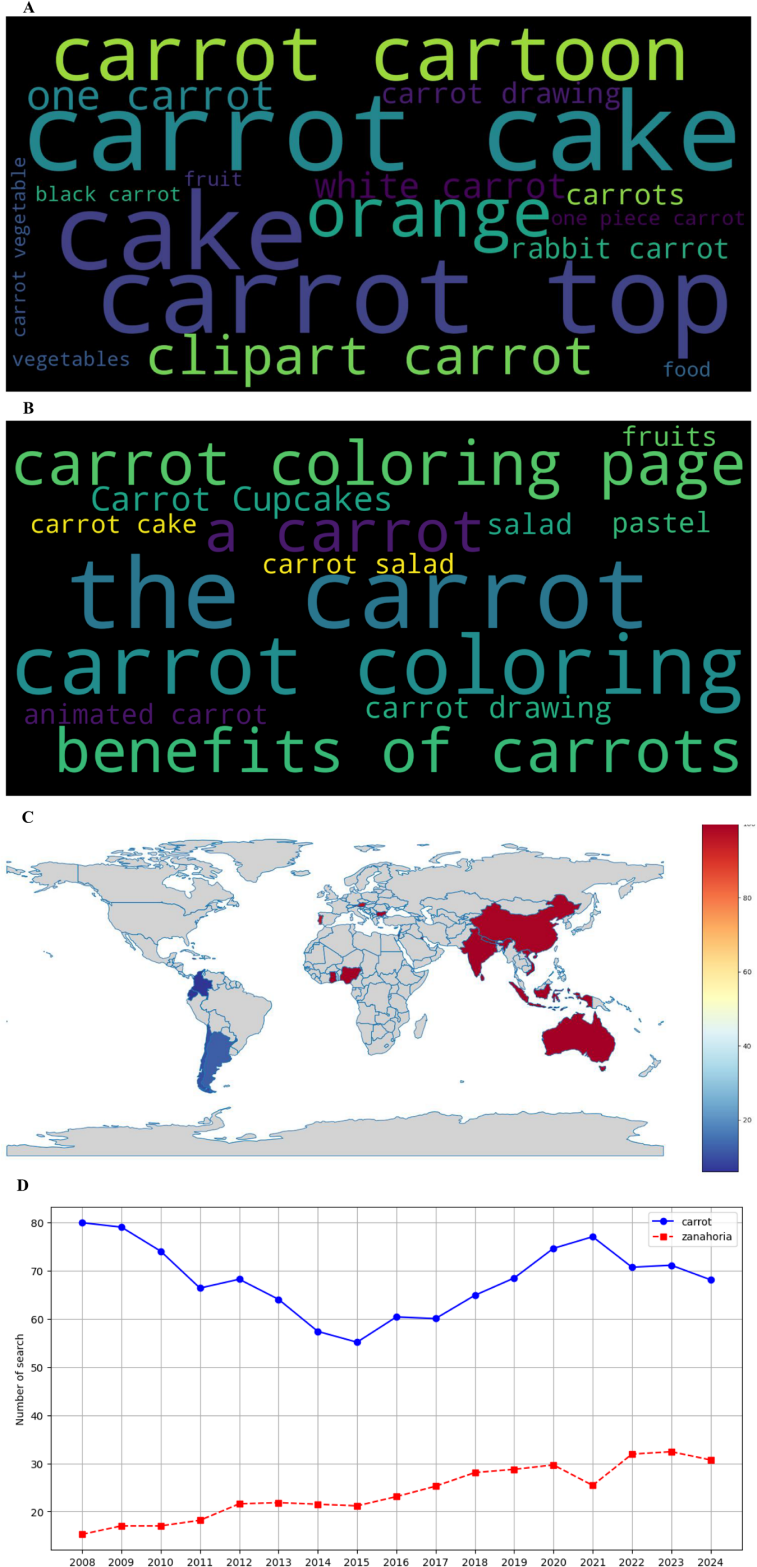
Search trend analysis on Google Images according to Google Trends. (A) Word cloud in English, (B) word cloud in Spanish, (C) geographic distribution map of searches, and (D) evolution of searches by year (2008–2024).

**FIGURE 9:**
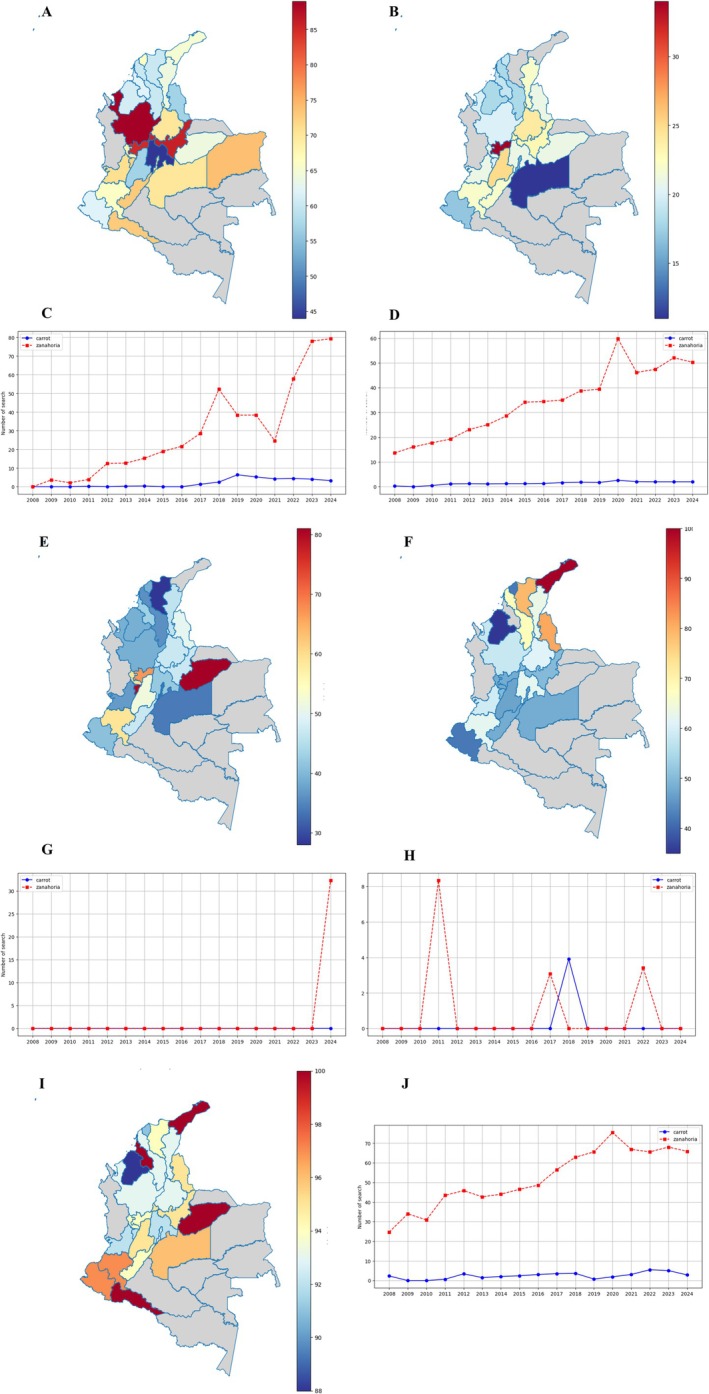
Search trends for “Zanahoria” across various Google Trends platforms in Colombia. (A) YouTube search trends, (B) web search trends, (C) evolution of searches per year (2008–2024) on YouTube, (D) evolution of searches per year (2008–2024) on web trends, (E) Google Shopping search trends, (F) Google News search trends, (G) evolution of searches per year (2008–2024) on Google Shopping, (H) evolution of searches per year (2008–2024) on Google News, (I) Google Images search trends, and (J) evolution of searches per year (2008–2024) on Google Images.

Image search analysis (Google Images, Figure 8) provided insights into visual preferences and educational needs. English searches (Figure 8A) emphasized identification (“types of carrots,” 19.3%) and practical applications (“carrot farm,” 14.7%), whereas Spanish searches (Figure 8B) focused more on culinary uses (27.9%). The geographic heatmap (Figure 8C) revealed particularly strong visual interest in South Asia and the Andean region. The temporal trend (Figure 8D) showed growing searches for colored varieties, increasing from 15% of image searches in 2015 to 28% in 2024 (Figure 9). YouTube searches for zanahoria (Figure 9C) increased steadily from 2011, peaking in 2013, 2017, and 2021, while carrot consistently had higher volumes. Antioquia, Cundinamarca, and Valle del Cauca showed the most interest (Figure 9A). On Google Web (Figure 9D), zanahoria searches peaked in 2015 and 2021, whereas carrot remained higher but declined slightly in 2023–2024. Andean regions (Boyacá, Nariño) led in searches (Figure 9B). Google Shopping (Figure 9G) had lower zanahoria activity, with peaks in 2018–2022, while carrot saw stronger interest. The Caribbean and Antioquia dominated (Figure 9E). Google News showed higher carrot coverage, especially in northern departments (Figure 9F). For Google Images (Figure 9J), zanahoria grew gradually, peaking in 2015–2022, but carrot maintained higher volumes. Antioquia and Atlántico led, while southern regions had minimal searches (Figure 9I).

### 3.3 Social Media Analysis: Reddit and YouTube

**FIGURE 10:**
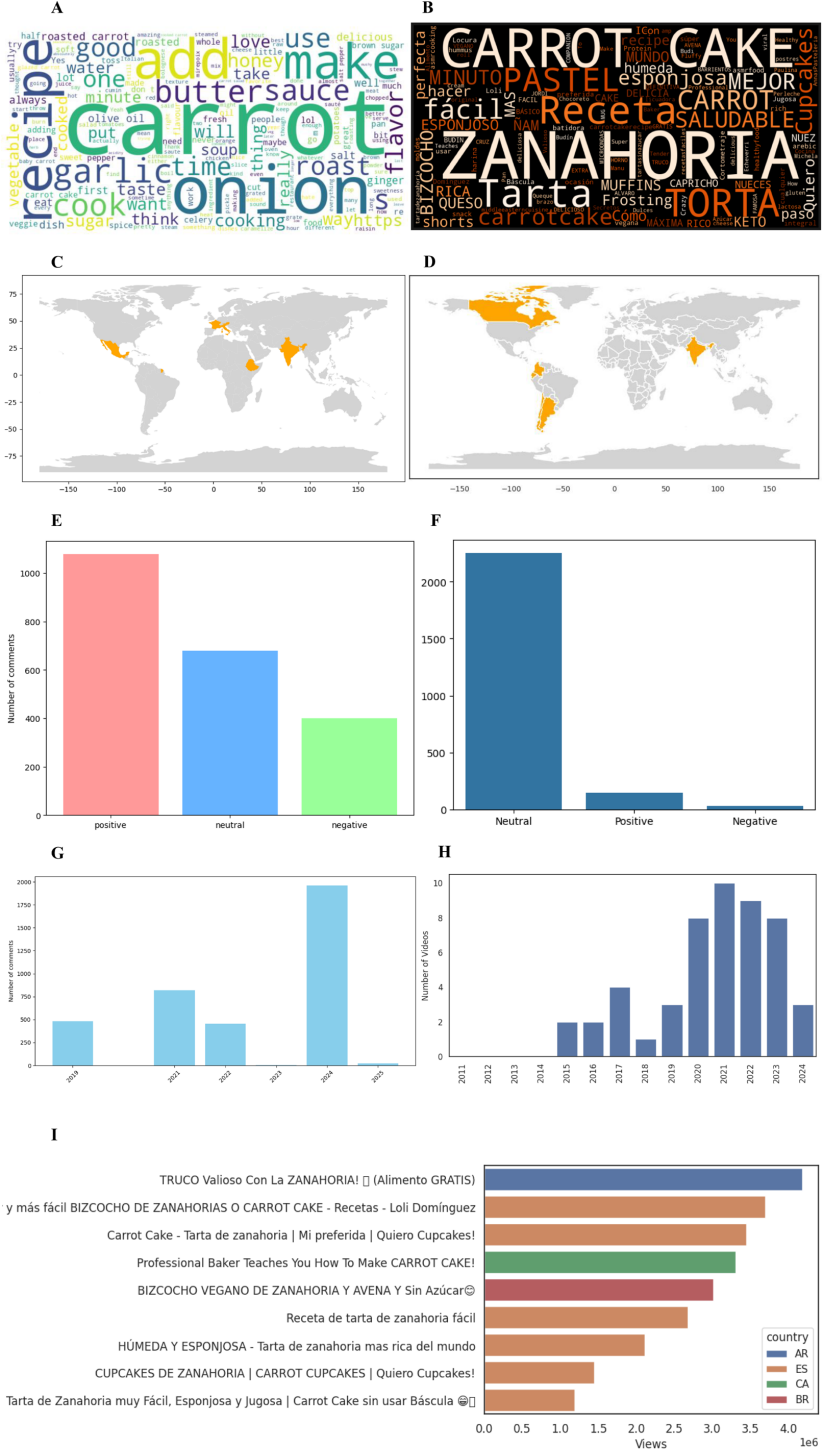
Search trends on YouTube and Reddit related to carrots. (A) Word cloud from Reddit, (B) word cloud from YouTube, (C) geographic distribution of mentions on Reddit, (D) geographic distribution of videos on YouTube, (E) sentiment analysis of comments on Reddit, (F) sentiment analysis of comments on YouTube, (G) temporal evolution of searches on Reddit, (H) temporal evolution of searches on YouTube, and (I) most viewed videos and their geographic distribution.

The examination of user‐generated content across social media platforms provided valuable insights into contemporary consumer perceptions and engagement patterns regarding carrot utilization. On Reddit (Figure 10A), lexical analysis of discussion threads revealed a predominant focus on culinary applications, with high‐frequency terms including foundational recipe components (“onion,” “garlic,” “butter”), preparatory verbs (“add,” “cook,” “make”), and cooking techniques (“roast,” “sauté”). This linguistic pattern demonstrates carrots' functional versatility as both a primary ingredient and flavor enhancer across diverse gastronomic preparations, ranging from savory dishes (evidenced by “soup,” “sauce”) to sweet applications (“honey,” “sugar”).

Sentiment distribution analysis (Figure 10E) quantified user attitudes, revealing predominantly positive sentiment (850 instances) characterized by descriptive terms like “flavorful,” “versatile,” and “nutritious.” Neutral comments (500 instances) typically contained factual information or recipe exchanges, whereas negative sentiment (< 300 instances) primarily addressed specific preparation challenges rather than inherent product qualities. Temporal analysis of comment volume (Figure 10G) showed peak engagement in 2024 (post‐pandemic period), with secondary spikes in 2021 corresponding to increased home cooking activity during global lockdowns. Geographic participation patterns (Figure 10C) identified Australia, Mexico, and India as the most active regions, whereas Latin American engagement remained comparatively limited, potentially reflecting platform adoption rates rather than actual interest levels.

YouTube content analysis (Figure 10B) demonstrated distinct platform‐specific consumption patterns, with Spanish‐language videos emphasizing baked goods (“Torta,” “Pastel,” “Bizcocho”) and preparation efficiency (“Fácil,” “Minuto,” “Microondas”). The prominence of dietary‐specific terminology (“Keto,” “Vegano,” “Integral”) reflects growing consumer demand for specialized nutritional content. Sentiment analysis of video comments (Figure 10F) showed 68% neutral (recipe queries, technique discussions), 32% positive (praise for recipes, nutritional benefits), and negligible negative sentiment, indicating strong audience receptivity.

Geographic distribution of video production (Figure 10D) revealed content creation concentrated in Western Hemisphere nations, with the United States (45 videos), Dominican Republic (20), and Brazil/Canada/Chile/India (10–20) as primary contributors. Temporal upload patterns (Figure 10H) showed sustained growth from 2015 to 2021, peaking during pandemic restrictions, followed by gradual stabilization at elevated baseline levels. Viral content analysis (Figure 10I) identified three dominant categories: traditional dessert preparations (42%), innovative culinary applications (33%), and agricultural/health education (25%), with maximum engagement achieved by videos combining visual appeal with practical utility.

### 3.4 Perception of the Multidimensional Quality Concept

**FIGURE 11:**
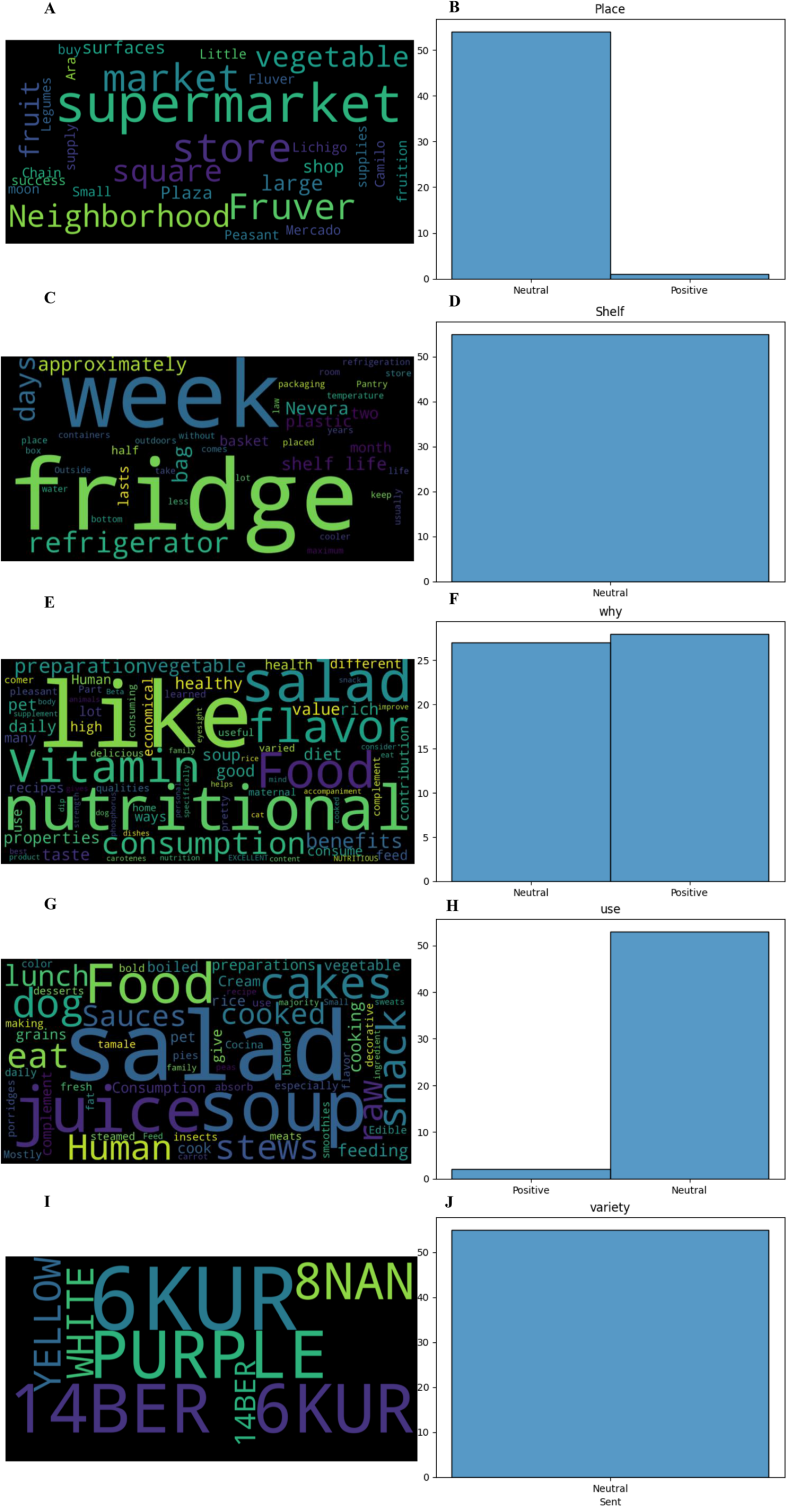
Quality parameters in the carrot value chain for consumers. (A) Word cloud of carrot purchasing locations, (B) sentiment analysis of carrot purchasing locations, (C) word cloud of storage and shelf life, (D) sentiment analysis of storage and shelf life, (E) word cloud of purchasing reasons, (F) sentiment analysis of purchasing reasons, (G) word cloud of carrot usage, (H) sentiment analysis of carrot usage, (I) word cloud of acceptance of new carrot cultivars, and (J) sentiment analysis of acceptance of new carrot cultivars.

Consumer purchasing patterns (Figure 11) reveal distinct preferences in retail channels, with supermarkets capturing (Figure 11A) 42% of carrot purchases, followed by local markets (31%) and specialty produce vendors (27%). Regional variations emerge through linguistic markers, where terms like “Fruver” and “neighborhood” reflect localized shopping behaviors. Storage practices (Figure 11C) demonstrate practical approaches to freshness preservation, with 89% of consumers refrigerating carrots for approximately 1 week. The majority (63%) maintain original packaging, whereas 37% transfer to dedicated containers, indicating a balance between convenience and optimal storage conditions.

**FIGURE 12:**
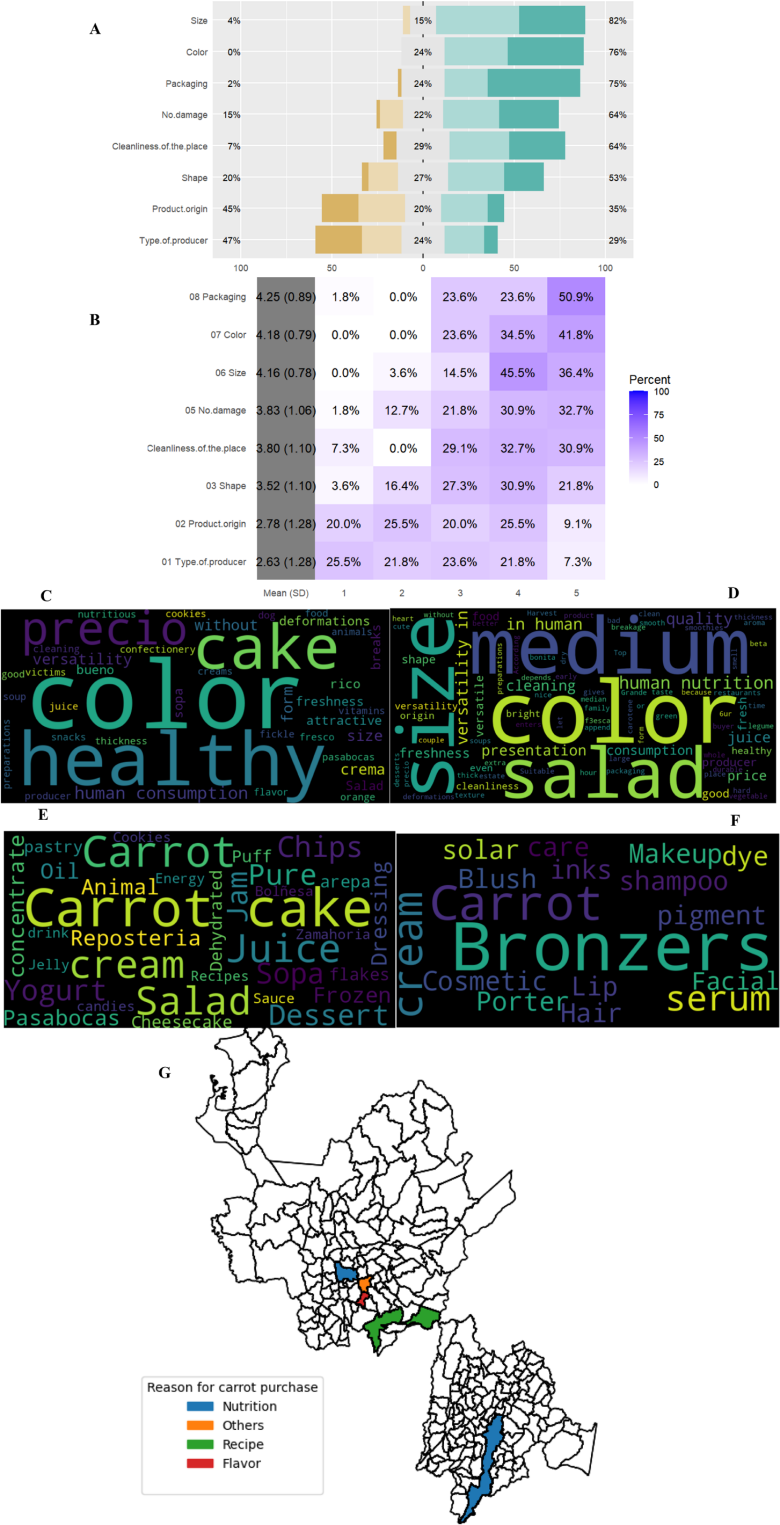
Analysis of responses from the Likert‐type survey on quality parameters in the carrot value chain. (A) Percentage distribution of responses by attribute, (B) heatmap of frequency distributions, (C) word cloud from perception surveys conducted in market plazas in Bogotá, (D) word cloud from perception surveys conducted in market plazas in Antioquia, (E) main terms associated with carrots in the food industry, (F) main terms associated with carrots in the cosmetic industry, and (G) distribution of reasons for purchasing carrots in municipalities of Cundinamarca and Antioquia.

Purchase motivations form three primary categories, with sensory qualities like flavor and texture driving 38% of decisions, nutritional benefits accounting for 35%, and practical factors such as price and availability influencing 27% of purchases (Figure 11E). Consumption patterns show carrots predominantly used (Figure 11G) in raw preparations (41%), particularly salads, followed by cooked dishes (33%) like soups and stews, and processed forms (18%) including juices. Notably, 8% of respondents reported using carrots for pet nutrition, demonstrating their cross‐species appeal.

Consumer openness to novel varieties presents interesting market dynamics (Figure 11I), with purchase intent for purple (62%) and yellow (58%) carrots surpassing traditional orange varieties (51%) among informed buyers. However, limited market availability currently restricts actual adoption rates. Sentiment analysis shows predominantly neutral responses (65%) shifting to positive (55%) when discussing specific quality attributes, suggesting that targeted information campaigns could enhance consumer engagement and product appreciation (Figure 11).

Quality evaluation criteria follow a clear hierarchy among consumers (Figure 12). Essential attributes commanding over 75% importance include size uniformity (82%), vibrant color (76%), and packaging integrity (75%). Secondary considerations (60%–74% importance) focus on product condition, particularly the absence of defects (64%) and firmness (61%). Production‐related factors like farm scale (47%) and organic certification (45%) rank as tertiary concerns, receiving less than 50% prioritization.

Geographic analysis (Figure 12) reveals significant regional differences in consumption patterns. In Antioquia, consumers emphasize aesthetic standards and fresh applications, whereas Bogotá shoppers prioritize economic factors and health benefits. Smaller municipalities demonstrate a stronger preference for traditional varieties and local flavors. Industrial applications show carrots serving dual purposes, with food processing accounting for juice production (54%), prepared salads (23%), and bakery ingredients (18%). In cosmetics, carrots feature prominently in skin care formulations (68%), along with hair treatments (22%) and sun protection products (10%), highlighting their versatile commercial value beyond nutritional applications.

## 4 Discussion

This study integrates bibliometric analysis, web trends, social media sentiment, and consumer surveys to reveal critical alignments and disparities between scientific research and consumer perceptions of carrot quality. Although the scientific literature emphasizes nutritional and functional attributes like carotenoid content (Šeregelj et al. 2020), consumers prioritize sensory and practical factors such as size, color, and culinary versatility. This divergence mirrors trends observed in other crops, where visual and tactile qualities often outweigh nutritional metrics in purchasing decisions (Péneau et al. 2007; Seljåsen et al. 2015). For instance, our meta‐analysis identified a genotypic variation in β‐carotene content, yet survey data showed that only 35% of consumers considered nutritional benefits a primary purchase driver, with most favoring uniform size (82%) and vibrant color (76%). This gap underscores the need for targeted communication strategies that bridge scientific insights with consumer priorities, perhaps by highlighting how nutritional attributes manifest in visible quality markers.

The findings provide actionable insights for stakeholders across the carrot value chain. For producers and breeders, the strong consumer preference for uniform size and vibrant color coupled with growing interest in non‐traditional varieties—suggests that breeding programs should prioritize these traits alongside nutritional enhancements, as seen in successful campaigns for purple carrots (da Silva Dias 2014). Marketers can leverage digital tools to bridge the gap between scientific research and consumer priorities; for instance, pairing claims about β‐carotene content with visual cues (e.g., “deep orange = more vitamin A”) could align health messaging with sensory appeal, a strategy proven effective in promoting functional foods (Sadler et al. 2019). Regional disparities in preferences, such as Bogotá's price sensitivity versus Antioquia's focus on freshness, call for tailored marketing campaigns—hyperlocal social media content or supermarket labeling that highlights region‐specific attributes. Policy‐makers could support underrepresented markets by funding digital literacy programs for small‐scale farmers, enabling them to access real‐time consumer trends via platforms like Google Trends, thus aligning production with demand. Additionally, the documented interest in carrot‐based cosmetics and processed products (e.g., juices) opens avenues for diversifying value‐added offerings, particularly in urban markets where convenience drives purchasing.

Geospatial and temporal analyses further contextualize these findings. Regions with robust agricultural research output, such as China and the U.S., correlated with higher scientific focus on carotenoid biosynthesis, whereas consumer interest in regions like Antioquia and Bogotá reflected localized cultural and economic factors. Antioquia's emphasis on freshness and size aligns with rural consumption patterns, whereas Bogotá's urban consumers prioritized price and health benefits a—dichotomy consistent with studies linking urbanization to heightened health awareness (Ayala‐Garay et al. 2016). Similarly, YouTube and Reddit data revealed spikes in searches for “purple carrot” and “carrot recipes” during the COVID‐19 pandemic, illustrating how global events can amplify interest in novel varieties and home‐based culinary uses. This trend parallels research by Sadler et al. (2019), who noted increased consumer experimentation with functional foods during health crises.

The growing acceptance of non‐traditional carrot varieties (e.g., purple and yellow carrots) highlights another critical intersection between research and consumer behavior. Scientific studies emphasize these varieties' antioxidant properties (da Silva Dias 2014), whereas social media sentiment analysis linked their appeal to visual novelty and perceived health benefits. However, market availability remains limited, suggesting an untapped opportunity for producers to align diversified offerings with consumer demand, as seen in successful campaigns for colored tomato varieties (Luby et al. 2014). The disconnect between research on postharvest preservation and consumer storage practices, where 63% of respondents retained original packaging despite evidence favoring dedicated containers, further underscores the need for practical, science‐backed consumer education.

Digital tools proved invaluable in capturing real‐time consumer sentiment, but platform limitations (e.g., reliance on Reddit and YouTube) may skew findings toward English‐ and Spanish‐speaking demographics. Future studies could expand to visually oriented platforms like Instagram to assess global trends more inclusively. Nonetheless, the consistency of our findings with broader literature on food quality perception (Allaire 2012) reinforces their validity. For example, the predominance of neutral‐to‐positive sentiment on social media echoes Medvedev et al.'s (2019) observations about Reddit as a barometer of consumer attitudes, whereas regional preference variations align with Crespi's (2015) work on geospatial consumption patterns.

While this study provides comprehensive insights into the multidimensional quality of carrots, certain limitations must be acknowledged. The temporal scope of the analysis (1990–2024 for bibliometrics; 2010–2024 for digital trends) may not fully capture long‐term shifts in consumer behavior or research priorities. Additionally, reliance on English‐ and Spanish‐dominated platforms (e.g., Reddit, YouTube) could introduce demographic biases, underrepresenting regions where other languages or platforms (e.g., Weibo, Jiji) predominate. The geographical focus on Colombia (primarily Antioquia and Bogotá) limits generalizability to global contexts, as regional socioeconomic and cultural factors heavily influence quality perceptions. Future research should expand to include more diverse linguistic and regional datasets, incorporating visually oriented platforms like Instagram or region‐specific channels to enhance representativeness. Further, controlled sensory studies with consumers could quantitatively validate the observed preferences for sensory attributes (e.g., color, size) versus nutritional claims. Economic analyses of non‐traditional varieties (e.g., purple, yellow carrots) would also clarify market viability and scalability, informing breeding and commercialization strategies. At last, longitudinal studies tracking the impact of digital literacy interventions on smallholder farmers' ability to leverage real‐time consumer trends could assess the practical efficacy of such policy recommendations. Addressing these gaps would strengthen the evidence base for aligning agricultural research with dynamic market and consumer needs.

## 5 Conclusions

- This study highlights a significant disconnect between scientific research on carrot quality, which emphasizes nutritional and functional attributes like carotenoid content, and consumer preferences, which prioritize sensory and practical factors such as size, color, and freshness. Although the meta‐analysis revealed substantial genotypic variation in β‐carotene content (5–80 mg/100 g), consumer surveys in Antioquia and Bogotá showed that only 35% of respondents considered nutritional benefits a primary purchase driver, compared to 82% who valued size uniformity and 76% who prioritized vibrant color. This misalignment underscores the need for targeted communication strategies that translate scientific insights into tangible quality markers (e.g., linking deep orange color to vitamin A content) to bridge this gap effectively.
- The findings reveal pronounced regional differences within Colombia. In Antioquia, consumers emphasized freshness and size, reflecting traditional market preferences, whereas Bogotá's urban consumers prioritized price and health benefits a—trend consistent with global patterns linking urbanization to health awareness. These disparities highlight the importance of localized marketing and production strategies. For instance, campaigns in Antioquia could highlight freshness through visual cues, whereas Bogotá's messaging might emphasize cost‐effectiveness and nutritional value. Such tailored approaches could enhance market alignment and consumer satisfaction.
- A key finding is the growing consumer acceptance of non‐traditional carrot varieties (e.g., purple and yellow carrots), driven by their visual appeal and perceived health benefits. Despite their higher antioxidant content (da Silva Dias 2014), these varieties remain underrepresented in markets. This presents an untapped opportunity for producers and breeders to diversify offerings, particularly targeting younger, health‐conscious demographics. Successful case studies, such as the introduction of colored tomato varieties, suggest that strategic promotion of these carrots could capture niche markets and enhance value‐chain profitability.
- The study demonstrates the utility of digital tools (Google Trends, social media sentiment analysis) in capturing real‐time consumer trends and regional disparities. For example, YouTube and Reddit data revealed spikes in searches for “purple carrot” during the COVID‐19 pandemic, aligning with broader shifts toward home cooking and functional foods. However, the reliance on English‐ and Spanish‐dominated platforms (e.g., Reddit, YouTube) may limit the generalizability of findings. Future research should incorporate visually oriented platforms like Instagram or region‐specific channels (e.g., Weibo) to ensure broader demographic representation.
- The geographical focus on Antioquia and Bogotá necessitates caution in extrapolating conclusions to other regions or global contexts. Although these areas provide valuable case studies, their socioeconomic and cultural specificities may not reflect broader trends. Future studies should expand to additional regions in Colombia and internationally to validate these findings. Additionally, integrating data from more diverse digital platforms and longitudinal surveys could further refine understanding of evolving consumer preferences.

## Author Contributions

**Paola Andrea Ospina‐Sanchez:** conceptualization (equal), data curation (equal), formal analysis (equal), funding acquisition (equal), investigation (equal), methodology (equal), project administration (equal), resources (equal), software (equal), supervision (equal), validation (equal), visualization (equal), writing – original draft (equal), writing – review and editing (equal). **Juan Camilo Henao‐Rojas:** conceptualization (equal), funding acquisition (equal), investigation (equal), methodology (equal), project administration (equal), resources (equal), supervision (equal), validation (equal), visualization (equal), writing – original draft (equal), writing – review and editing (equal). **Joaquín Guillermo Ramírez‐Gil:** conceptualization (equal), formal analysis (equal), investigation (equal), methodology (equal), resources (equal), software (equal), supervision (equal), validation (equal), visualization (equal), writing – original draft (equal), writing – review and editing (equal).

## Ethics Statement

This study was conducted under a strict ethical compliance process, adhering to *Habeas Data* regulations. For each survey conducted, informed consent was obtained, and participants were informed that their data would be handled confidentially. All procedures followed ethical guidelines to ensure privacy, transparency, and responsible data management.

## Conflicts of Interest

The authors declare no conflicts of interest.

## Data Availability

The current study is available from the corresponding author upon reasonable request.
